# Aging of hippocampal neurogenesis and soy isoflavone

**DOI:** 10.18632/oncotarget.13534

**Published:** 2016-11-23

**Authors:** Jun Yamada, Shozo Jinno

**Affiliations:** Department of Anatomy and Neuroscience, Graduate School of Medical Sciences, Kyushu University, Fukuoka, Japan

**Keywords:** daidzein, adult neurogenesis, hippocampus, aging, Neuroscience

Throughout life, production of new neurons (granule cells) continues in the hippocampus of mammals including humans. Adult-generated granule cells play an important role in regulation of hippocampus-dependent cognitive functions and emotional behavior [1]. Each process of adult hippocampal neurogenesis is controlled by a variety of extrinsic and intrinsic factors. For instance, physical restraint, immobilization, foot shock and aging, have been shown to decrease production of granule cells. Especially, aged animals with preserved spatial memory exhibit a higher levels of adult hippocampal neurogenesis, while those with spatial memory deficits show a reduction in production of new granule cells. Experimental enhancement of adult hippocampal neurogenesis counteracts cognitive decline in aged animals. Furthermore, defects in adult hippocampal neurogenesis are suggested to be involved in cognitive dysfunction clinically observed in aged people. It is thus expected that the manipulation of adult hippocampal neurogenesis may help to rescue cognitive impairment in elderly people.

Estrogens influence not only ovulation and reproductive behavior but also affect cognitive functions via estrogen receptors (ERs) [2]. In non-demented female populations, reductions of endogenous estrogens following menopause are often linked to cognitive deficits, with such decrements being reliably reversed by estrogen replacement. However, many women hesitate to take estrogens because they are afraid it might cause breast and endometrial cancer. Thus, there is now a need for safe and acceptable estrogen substitutes. Phytoestrogens are plant derived compounds that exhibit biological functions comparable to estrogens. Although phytoestrogens consist of a variety of compounds, they can largely be divided into three classes: isoflavones, lignans, and coumestans. Recent studies suggest that dietary and supplemental soy isoflavones can offer some relief for menopausal symptoms including hot flashes and vaginal dryness [3]. However, the mechanisms underlying phytoestrogen action on the brain remain to be elucidated.

To get insight into the potential benefits of soy isoflavones on cognitive dysfunction following menopause, we have focused on the effects of daidzein on the adult hippocampal neurogenesis in middle-aged (12-month-old) female mice [4]. Animals received daily intraperitoneal injections of daidzein for four weeks, and the cells at specific stages of neurogenesis were presumptively defined using molecular markers. Administration of daidzein increased the spatial densities of late transient amplifying progenitors (TAPs), neural progenitors, and immature granule cells. However, the densities of primary progenitors and early TAPs were not affected by daidzein. These findings may shed new light on the “critical period” hypothesis of hormone therapy [5]: the use of estrogen therapy for middle-aged women protects against cognitive impairment, whereas the initiation of estrogen replacement in late life has only deleterious effects. Although it is rather difficult to obtain clinically useful information from our research, we suggest that cell-type specific enhancement of adult hippocampal neurogenesis by daidzein may support the “critical period” hypothesis of hormone therapy. Namely, daidzein cannot promote new cell production in the adult hippocampus under the condition where primary progenitors are depleted.

How daidzein promotes adult hippocampal neurogenesis is an intriguing question. In this regard, our results have shown that cell proliferation and dendritic length are increased by daidzein. The estrogen signaling pathway includes both a nucleus-initiated (genomic) response and a membrane-initiated (non-genomic) cascade. Estrogens and brain-derived neurotrophic factor (BDNF) activate the same effectors and exert similar actions, such as promotion of proliferation and neurite growth. Specifically, daidzein increases the expression levels of BDNF through its action on nuclear ERs. This process is considered to underlie the increase in cell proliferation following daidzein administration. It has also been suggested that membrane-associated ERs may be responsible for the action of daidzein on neuronal maturation, such as neurite growth. Our findings regarding the positive effects of daidzein on proliferation of progenitor cells and dendritic growth of new granule cells indicate that daidzein may act on both nucleus- and membrane-associated ERs.

Our recent study also indicates that adult hippocampal neurogenesis is promoted by daidzein more effectively in the dorsal region than in the ventral region. The hippocampus of mammals is genetically and functionally differentiated along its longitudinal axis [6]. In adult rodents, the dorsal hippocampus plays a preferential role in cognition and memory, and the ventral hippocampus is implicated in emotional processing. Dorsoventral difference has previously been identified in the adult hippocampal neurogenesis: rates of new cell production are higher in the dorsal than in the ventral regions in young adult male mice [7]. Interestingly. the expression patterns of ERs in the hippocampus are different between dorsal and ventral regions. Together, these results indicate that region-specific effects of daidzein may be associated with the dorsoventral difference in daidzein receptors (ERs and/or non-ERs), and also suggest that daidzein may be beneficial in rescuing cognitive deficits, but less effective for treatment of emotional disturbances. Our observations provide a new insight into how soy isoflavone daidzein can counteract cognitive menopausal symptoms via enhancement of adult hippocampal neurogenesis.

## References

[1] Goncalves JT (2016). Cell.

[2] Arevalo MA (2015). Nat Rev Neurosci.

[3] Franco OH (2016). JAMA.

[4] Yamada J (2016). Neuropharmacology.

[5] Sherwin BB. (2009). Nat Rev Endocrinol.

[6] Strange BA (2014). Nat Rev Neurosci.

[7] Hippocampus. Jinno S. (2011).

